# The displaced polymer clamp entered the duodenum causing an abscess: A case report

**DOI:** 10.1097/MD.0000000000035783

**Published:** 2023-10-27

**Authors:** Yong Qiang Yang, Wen Bin Gou

**Affiliations:** a Digestive Endoscopy Department, People’s Hospital of Wanning, Wanning, Hainan, P. R. China; b Department of Pathology, People’s Hospital of Wanning, Wanning, Hainan, P. R. China.

**Keywords:** abscess, gastroscopy, Hem-o-lok ligation clips, perforation

## Abstract

**Rationale:**

The Hem-o-lok clip, made from a nonabsorbable polymer, and its predecessor the metal ligation clip have been used widely for laparoscopic or robot-assisted surgery to ligate the cystic duct after a cholecystectomy, to ligate the appendix after an appendectomy, or control hemorrhage or on occasion to occlude a fistula or enterotomy. Displacement of these ligation clips to distant sites is an extremely rare complication in clinical practice.

**Patient concerns:**

The patient is a 67-year old female who sought medical attention for 3 days due to worsening intermittent upper abdominal pain and poor appetite. Gastroscopy showed both an ulcer and the presence of a foreign object embedded in the anterior wall of the duodenal bulb, consistent with what looked like a polymer-based ligation clip. After removal of the foreign body, which turned out to be a remnant of the polymer clip, no further pus was seen, but fresh granulomatous tissues were seen at the base.

**Diagnoses:**

a polymer-based clip-induced duodenal bulb erosion with a local contained enterically draining abscess.

**Interventions:**

The patient recovered after removing foreign bodies under gastroscopy and receiving anti infection treatment.

**Outcomes:**

The patient recovered after removing foreign bodies under gastroscopy and receiving anti infection treatment.

**Lessons:**

In laparoscopic cholecystectomy, attention should be paid to the correct surgical techniques, possibly by decreasing the number of such clips used or considering use of absorbable clips, ligature wires, ligation with absorbable suture material, or ultrasonic resection, all of which can be used for clipless cholecystectomy.

## 1. Introduction

The Hem-o-lok clip, made from a nonabsorbable polymer, and its predecessor the metal ligation clip have been used widely for laparoscopic or robot-assisted surgery to ligate the cystic duct after a cholecystectomy, to ligate the appendix after an appendectomy, or control hemorrhage or on occasion to occlude a fistula or enterotomy. Displacement of these ligation clips to distant sites is an extremely rare complication in clinical practice. Many previous case reports focused on displacement of metallic clips used to ligate the cystic duct during cholecystectomy into the digestive tract, bile duct, and urinary tract; most cases have occurred several days or months after their application. This process of displacement can on occasion lead to local bleeding or bile leakage if placed in the biliary tree, and the presence of a nonabsorbable foreign body in the lumen of the biliary tree can also lead to the development of choledocholithiasis. Therefore, metallic clips have been replaced by polymer-based ligation clips, which, because they are made of a polymer substance, appear to have a better safety profile in clinical practice. No prior report to the best of our knowledge exists of duodenal erosion by a displaced polymer-based ligation clip after a cholecystectomy. In this report, we describe our diagnosis and treatment of a patient with a polymer-based clip-induced duodenal bulb erosion with a local contained enterically draining abscess.

## 2. Case presentation

The patient was a 67-year-old female who sought medical attention at the emergency department of People’s Hospital of Wanning, China, on February 7, 2022 due to exacerbation of intermittent epigastric pain and poor appetite for 3 days. The patient had experienced intermittent, dull epigastric pain for the previous 6 months, which would resolve with antacids. In the last 3 days, epigastric pain worsened progressively and became intolerable. Fever, nausea, vomiting, and abdominal distension were absent. Past medical history was pertinent for hypertension and previous appendectomy and laparoscopic cholecystectomy (LC), but no history of duodenal ulcer or a prior gastroscopy. On physical examination, she was afebrile and without jaundice. Blood pressure and heart rate were normal. The abdomen was flat without evidence of peritonitis.

**Figure 1.**
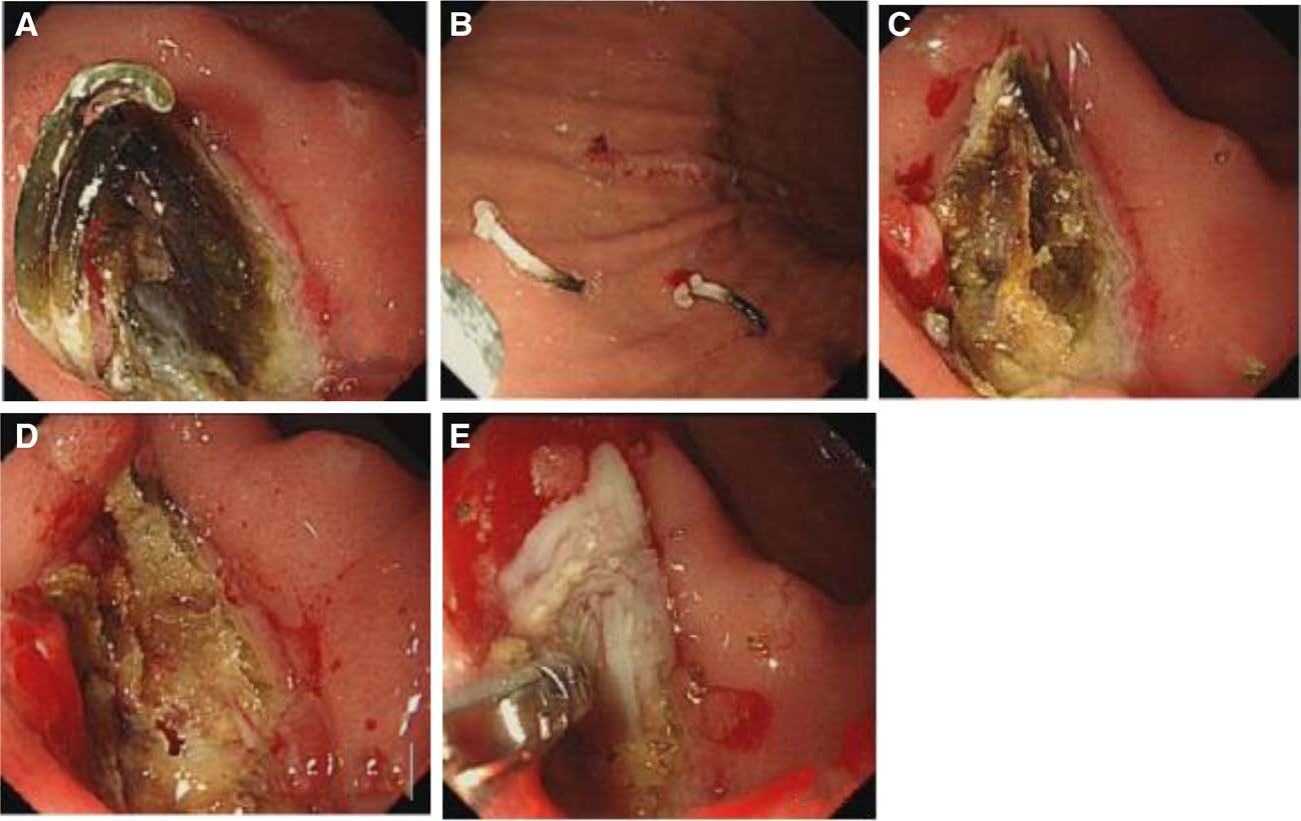
(A) Duodenal bulb foreign body, (B) ligation clip removal, (C) foreign body at base of ulcer, (D) opening at base of ulcer, and (E) Pus discharge seen after opening.

**Figure 2.**
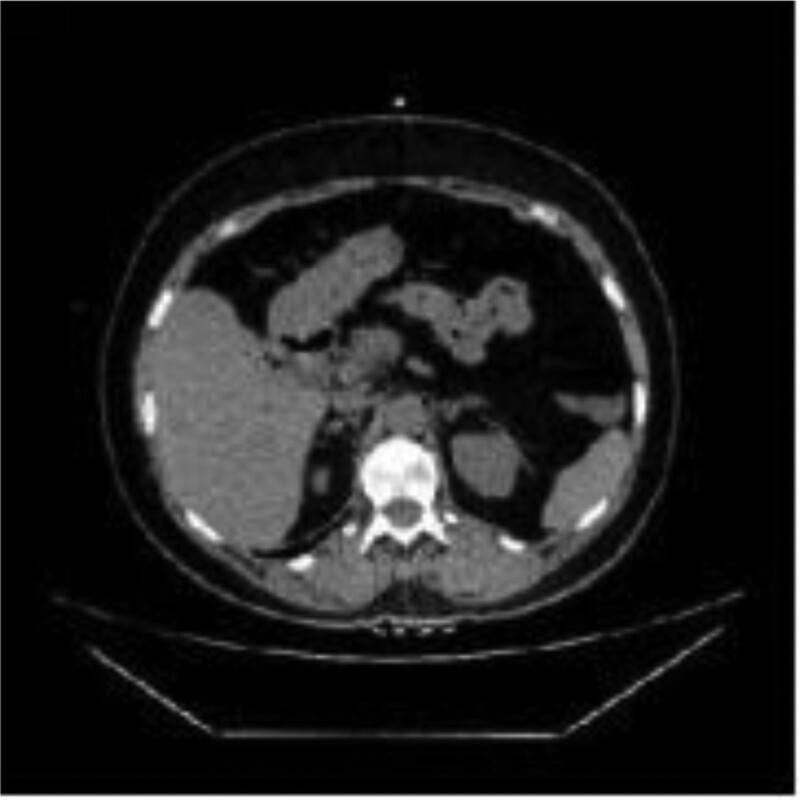
CT showing local periduodenal gas accumulation after gastroscopic removal of polymer-based ligation clip foreign body. CT = computed-tomography.

**Figure 3.**
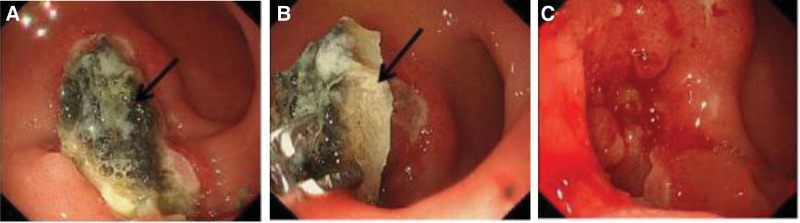
(A) Reexamination of site of duodenal erosion with residual foreign body. Arrows point to residual flaky foreign matter. (B) Foreign body removal using foreign body forceps. Arrows point to the residual foreign bodies, which was in the duodenal bulb mucosa, and the black surface was caused by acid corrosion. (C) Fresh granulomatous tissues seen at base of foreign body.

No apparent abnormalities were observed from routine blood screening, but because of her complaints, a contrast-enhanced abdominal computed-tomography was obtained and showed that the morphology of the antral wall of the stomach and proximal duodenum was abnormal and had mild enhancement, suggesting inflammation. She was admitted, and gastroscopy showed both an ulcer (1.2 cm × 1.5 cm in size) and the presence of a foreign object embedded in the anterior wall of the duodenal bulb, consistent with what looked like a polymer-based ligation clip (Fig. 1A and B). This clip was removed, after which a large volume of pus discharged from the base of where the foreign object was removed (Fig. 1C–E). Because it was not clear if the abdominal cavity was penetrated, a computed-tomography showed a localized encapsulated abscess with small amounts of gas accumulation adjacent to the duodenal bulb. (Fig. 2). At this point, the diagnosis was made of displacement of this polymer-based ligation clip from her previous LC with penetration into the anterior duodenum resulting in a contained, enterically draining perforation. The patient’s abdominal pain resolved, and she was treated with rabeprazole to inhibit gastric acid secretion and sulfoaluminum to protect the duodenal mucosa for 30 days after which her diet was advanced. She was not treated with antibiotics because the abscess was controlled. Although the patient did not develop worrisome abdominal pain, some element of abdominal discomfort was present after overeating. Therefore, 6 weeks after her original presentation, she underwent a gastroscopy which showed persistence of a duodenal bulb ulcer and a residual foreign body (Fig. 3A). After removal of the foreign body, which turned out to be a remnant of the polymer clip (Fig. 3B), no further pus was seen, but fresh granulomatous tissues were seen at the base (Fig. 3C). After fasting for 1 day with antacid and nutritional support treatment, the patient had no complaints, and she has remained asymptomatic for 3 months.

## 3. Discussion

In 1987, LC was introduced and rapidly revolutionized cholecystectomy clinical practice because of the short duration of hospitalization, small incisions, few complications, fast postoperative recovery, and high surgical safety; and thus, LC became the standard of care for treating gallbladder and common bile duct calculi and other benign gallbladder lesions.^[1,2]^ To facilitate laparoscopic ligation of the cystic duct, laparoscopic-applied clips were developed, first made of metal and then of a putatively less-reactive polymer. The polymer-based clips, popularized as Hem-o-lok ligation clips, are made from nonabsorbable polymers with low tissue reactivity, do not cause any tissue cutting, and have a wide ligation area. Compared with the traditional metallic clips, these polymer-based clips do not produce radiopaque artifacts during radiologic examinations, nor do they detach or displace easily after placement. Therefore, these polymer-based clips are widely used in many practices for laparoscopic and robot-assisted operations in China.^[3]^

In clinical practice, displacement of these ligation clips is rarely seen. The first case of ligation clip displacement was reported in *Endoscopy* in 1992.^[4]^ In the past, the probability of displacement was considered to be greater for metallic clips than for polymer-based ligation clips. Displacement occurs most commonly in the biliary system, because they are used most frequently there; displacement can lead to bile leakage, development of bile duct calculi, obstructive jaundice, and even acute pancreatitis.^[5]^ Reports of duodenal bulb perforation caused by displaced ligation clips from a prior cholecystectomy, however, are rare.^[6,7]^ The exact process by which a clip is displaced to the duodenum is still debated; however, it is generally believed that clip displacement is related to cases in which the ligated cystic duct lies anatomically near the duodenum. Other potential causes of displacement may be related to non-standardized use of electrosurgical equipment causing local tissue damage, postoperative local tissue inflammation, local mucosal ischemia and necrosis, foreign body rejection reaction, and mechanical damage caused by the ligation clip.^[8]^ Clip displacement have been reported to cause choledocholithiasis, pancreatitis, duodenal ulcers, and biliary-colonic fistulas. Radiologic examination cannot differentiate between a displaced ligation clip and calculi very well because clips and about 80% of gallstones are radiopaque. Regarding when clip displacement causes complications, it is generally believed that the clips should be removed after the diagnosis is confirmed.^[9]^

In our patient, we maintain that the displaced clip resulted in local tissue inflammation and subsequent erosion into the duodenum. Repeated irritation and local infection caused a local foreign body reaction that resulted luckily in a contained enterically draining abscess resulting in severe pain. Initially, we used forceps to remove the displaced clip under gastroscopy, but we were not able at that time to appreciate another foreign body at the base of the perforation until repeat gastroscopy 6 weeks later because of persistent symptoms.

Clinicians should pay sufficient attention to patients with persistent complaints even long after a prior LC. Although clip displacement is quite rare, this diagnosis should at least be entertained in patients with recurrent choledocholithiasis, jaundice, pancreatitis, or unexplained abdominal pain. In appropriate clinical settings, conservative treatment can be considered for asymptomatic clip displacement.^[10]^

## 4. Conclusion

In conclusion, attention should be paid to correct operative techniques during laparoscopic cholecystectomies, possibly by decreasing the number of such clips used or considering use of absorbable clips, ligature wires, ligation with absorbable suture material, or ultrasonic resection, all of which can be used for clipless cholecystectomy.

## Acknowledgments

We thank the patient and their family for their participation in this study. There is no conflict of interest for all authors.

## Author contributions

**Writing – original draft:** Yong Qiang Yang.

**Writing – review & editing:** Wen Bin Gou.
